# Dr. Cartwright on “Drapetomania”

**Published:** 1854-12

**Authors:** 


### EDITORIAL DEPARTMENT.

---

*Dr. Cartwright on "Drapetomania."*—The Georgia Blister and Critic is a monthly, published at Atlanta, Georgia, and is especially devoted and foreordained "to the exposure of quackery, the development of Southern medicine, and the diseases and physical peculiarities of the Negro race."

It is edited in a most masterly manner by Dr. H. H. Ramsey, the gentleman who had, some years ago, such a marvellous number of obstetrical cases. For any further information as to the "Blister," its objects and character, we refer all inquiring minds to the editor of the New Jersey Medical Reporter, a journal for whose opinion we have always had a sincere regard, ever since (*Eheu! fugaces! fugaces! labuntur anni, etc.*, &c.,) some years agone it gave us our *first* and most delicious puff, for an article, our first essay, in which we proved—we have forgotten what we did prove.

Our purpose in this formal introduction, is to give due importance to an article recently published in its pages by Dr. Samuel Cartwright, of New Orleans. Characterized by the same cautious induction and logical accuracy which ever attend the literary efforts of that gentleman, it deserves the careful consideration of the medical philosopher, the anatomical statesman, and the benighted Saratoga convention.

Those of our readers who are in the habit of referring to Cullen's Nosology for the definition of diseases, will find no mention there of *Drapetomania.* The ignorance of the ancients was surprising, and we need but refer to Drapetomania as an evidence of the progressive spirit of the age in which we live.

Dr. Cartwright has conferred this name, Drapetomania, upon a disease peculiar to the South, and which is, we believe, entirely confined to that section, and only manifested at the North in certain analogous if not identical forms, which we shall have occasion to mention when we have given our readers time for the perusal of the following extract from Dr. Cartwright's able article:

"*Drapetomania, or the Disease causing Slaves to run away.*— Drapetomania is from * * * * * * a runaway slave, and * * * *mad or crazy.* It is unknown to our medical authorities, although its diagnostic symptom, the absconding from service, is well known to our planters and overseers, as it was to the ancient Greeks, who expressed by the single word * * * * * the fact of the absconding, and the relation that the fugitive held to the person he fled from. I have added to the word meaning runaway slave, another Greek term, to express the disease of the mind causing him to abscond. In noticing a disease not heretofore classed among the long list of maladies that man is subject to, it was necessary to have a new term to express it. The cause, in the most of cases, that induces the negro to run away from service, is as much a disease of the mind as any other species of mental alienation, and much more curable, as a general rule. With the advantages of proper medical advice, strictly followed, this troublesome practice that many negroes have of running away can be almost entirely prevented, although the slaves be located on the borders of a free state, within a stone's throw of the abolitionists. I was born in Virginia, east of the Blue Ridge, where negroes are numerous, and studied medicine some years in Maryland, a slave state, separated from Pennsylvania, a free state, by Mason & Dixon's line—a mere air line, without wall or guard. I long ago observed that some persons, considered as very good, and others as very bad masters, often lost their negroes by their absconding from service; while the slaves of another class of persons, remarkable for order and good discipline, but not praised or blamed as either good or bad masters, never ran away, although no guard or forcible means were used to prevent them. The same management which prevented them from walking over a mere nominal unguarded line, will prevent them from running away anywhere.

"To ascertain the true method of governing negroes, so as to cure and prevent the disease under consideration, we must go back to the Pentateuch, and learn the true meaning of the untranslated term that represents the negro race. In the name there given to that race is locked up the true art of governing negroes in such a manner that they cannot run away. The correct translation of that term declares the Creator's will in regard to the negro; it declares him to be the submissive knee-bender. In the anatomical conformation of his knees, we see '*genu flexit*' written in the physical structure of his knees, being more flexed or bent, than any other kind of a man. If the white man attempts to oppose the Deity's will, by trying to make the negro anything else than '*the submissive knee bender*,' (which the Almighty declared he should be,) by trying to raise him to a level with himself, or by putting himself on an equality with the negro; or if he abuses the power which God has given him over his fellow man, by being cruel to him or punishing him in anger, or by neglecting to protect him from the wanton abuses of his fellow servants and all others, or by denying him the usual comforts and necessaries of life, the negro will run away; but if he keeps him in the position that we learn from the scriptures he was intended to occupy, that is, the position of submission, and if his master or overseer be kind and gracious in his bearing toward him without condescension, and at the same time ministers to his physical wants and protects him from abuses, the negro is spell-bound, and cannot run away. '*He shall serve Japheth; he shall be his servant of servants*;' on the condition above-mentioned—conditions that are clearly implied, though not directly expressed. According to my experience, the 'genu flexit'—the awe and reverence, must be exacted from them, or they will despise their masters, become rude and ungovernable, and run away. On Mason & Dixon's line, two classes of persons were apt to lose their negroes; those who made themselves too familiar with them, treating them as equals, and making little or no distinction in regard to color; and, on the other hand, those who treated them cruelly, denied them the common necessaries of life, neglected to protect them against the abuses of others, or frightened them by a blustering manner of approach, when about to punish them for misdemeanors. Before negroes run away, unless they are frightened or panic-struck, they become sulky and dissatisfied. The cause of this sulkiness and dissatisfaction should be inquired into and removed, or they are apt to run away or fall into the negro consumption. When sulky and dissatisfied without cause, the experience of those on the line and elsewhere, was decidedly in favor of whipping them out of it as a preventive measure against absconding or other bad conduct. It was called whipping the devil out of them.

"If treated kindly, well fed and clothed, with fuel enough to keep a small fire burning all night, separated into families, each family having its own house—not permitted to run about at night; or to visit, or to use intoxicating liquors, and not overworked or exposed too much to the weather, they are very easily governed — more so than any other people in the world. When all this is done, if any one or more of them at any time, are inclined to raise their heads to a level with their master or overseer, humanity and their own good require that they should be punished until they fall into that submissive state which it was intended for them to occupy in all after time, when their progenitor received the name of Canaan, or 'submissive knee-bender.' They have only to be kept in that state, and treated like children, with care, kindness, attention and humanity, to prevent and cure them from running away."

Having always lived in the neighborhood of latitude 44°, and in the immediate vicinity of large bodies of fresh water, we have had only occasional opportunities for witnessing the disease thus graphically described. Such cases as have come under our notice we have observed with as near an approximation to the precepts of "What to observe at the bedside," as was convenient or possible in consideration of the fact that the unfortunate patients never went to bed at all, but were seemingly possessed by an obstinate propensity to travel northward. We have devoted not a little time to the elucidation of this singular morbid phenomenon, and, after a series of experiments upon the lower animals, we have arrived at the following conclusions:

*Primus.* The vital electricity not unfrequently becomes "detached," and occasions singular muscular movements,* such as writing sermons, speeches, and spiritual communications, without the exercise of volition.

*Secundus.* This "vital electricity" in its "*attached*" condition, determines the movements of men in a direction contrary to the diurnal revolutions of the earth. Hence "westward the course of empire takes its way," simply in accordance with this antagonism of attached vital electricity, and the motion of the globe upon its own axis.

*Tertius.* In the "detached" form of vital electricity this contrariety of action is lost, the nervous erythism of the human body is thrown into relations with the magnetic pole, and thus "drapetomania" is produced; the

---

* Vide Boston Medical and Surgical Journal, vol. xlix.

proclivity of the patient being always on a line parallel with the magnetic needle, thus directing his footsteps northward.

In laying down the above propositions we are aware that we have taken a tremendous step in advance of the age; but are consoled by the thought that future generations will acknowledge their scientific accuracy. Leaving them to their own defence, we pass on to the description of a symptom of Drapetomania not mentioned by Dr. Cartwright.

The disease does not exist north of the great chain of lakes—or rather its distinguishing symptom is lost—the tendency to hyperborean progression disappearing, the nervous system resuming its natural status, and the westward tendency frequently returning. The system, however, never entirely recovers from the shock, and consequently a disposition to return to the southward is never manifested. The patient, upon arriving at the northern shore, has a sudden convulsion of the inferior extremities, known as "Juba," or "Heel and toe," and on coming out of this convulsive action is to all appearance well. We regret that we have never had any opportunities for the treatment of Drapetomania, but we are still able to cull from analogy some facts which go to confirm the curative means proposed by Dr. Cartwright.

A tendency to flight from magisterial correction is not unfrequently manifested by school boys at the north, leading them to desert the roof devoted to the caquirement of the rudiments of science, and spend their time in migratory and predatory incursions among the fields and orchards of the rural districts. This disease has been named "Effugium discipulorum," the designation being taken from the Latin on account of a defective knowledge of Greek on the part of its inventor.

This complaint is strikingly analogous to Drapetomania, and we are gratified to find that the treatment recommended by Dr. Cartwright in the latter, is the same as that which has, since the days of Solomon, been found so efficacious in Effugium Discipulorum. Dr. Cartwright says that "the experience of those on the line, and elsewhere, was decidedly in favor of whipping them out of it as a preventive measure against absconding, or other bad conduct." The same means have been sanctioned by ages of experience in Effugium Discipulorum; thus confirming the allied nature of the two diseases and the correctness of Dr. C.'s hypothesis. H.

